# Nervous Diseases

**Published:** 1905-11-18

**Authors:** 


### NERVOUS DISEASES.

**Mental Symptoms in Certain Nervous Diseases.**—The mental symptoms in *neurasthenia*, a disease which has been classed by Kraft-Ebing as a neuropsychosis, are often more prominent than the physical signs. The most important have been thus summarised by E. L. Hunt[15]: Mental irritability and a carping fault-finding disposition, especially after undue excitement or fatigue, an unpleasant peculiarity from which many who consider themselves healthy are not exempt; depression, variable and often deep; weakened power of attention and slow cerebration—the neurasthenic cannot think brightly and connectedly nor fix his attention for long; introspection, especially as regards the physical functions and well-being; impaired memory, especially for recent events; impulses, imperative ideas, or obsessions, which must be attended to or the patient cannot rest. They may be simple, as returning to make sure the door is bolted, or take the more serious forms of indecision, causeless anxiety, or phobia. The commonest phobias are the fear of closed places, of disease, and of contamination. Often there is but a step between these and actual insanity. The neurasthenic, however, tries to resist his morbid tendencies, and hence arises one contrast between this condition and hysteria, in which the will-power is in abeyance and the symptoms are not resisted, and ultimately are even encouraged. In a recent clinical lecture on hysteria, Hale White[16] turns to the views enunciated by Myers in his book on "Human Personality" for a possible explanation of the complex and difficult problems presented by this disease. Briefly, Myers supposes two selfs—a supraliminal self, a self above the threshold, and a subliminal self, a self below the threshold. The former, the higher, supraliminal self is our ordinary self, the only one of which in health we are aware. But in the lower subliminal self mental processes go on of which we are not ordinarily cognisant. If in hysteria the supraliminal self be supposed to be wholly or partly in abeyance, the lower subliminal self may be regarded as rising unchecked into prominence and dominating the personality. The restraints of the higher self will then be gone. Such a view may help to explain some hysterical symptoms such as the anæsthesias and contracted visual fields, of which the patient may be wholly unaware.

In *cerebral tumour* much variety of symptom is seen, both physical and mental dependent on the nature, size, and especially the site of the tumour, but some mental change is common. It was noted in fifty-eight of sixty-four cases of apparently uncomplicated growth observed by O. C. Knapp.[17] Most of these showed simple mental failure and dullness; fifteen had actual delirium, and in seven there was decided mental confusion. It is interesting to observe that in the only case of tumour of the cerebrum without mental symptoms the growth was situated in the frontal lobe. The general nature of the mental disturbances suggested a toxic origin. In this series of cases no instance of the state of extreme exhilaration, known as *Hitzelsucht*, occurred. In another case reported by Brownrigg,[18] that of a man in whose frontal lobe a necrotic mass the size of a hen's egg was found, which had probably existed for months, mental symptoms and incapacity for work only appeared a fortnight before death.

The mental disturbances, associated with some cases of *multiple neuritis*, were specially described by Korsakoff in 1887. Two instances, presumably of alcoholic origin, in which this symptom complex was present, have been reported by F. R. Sims.[19] In each the autopsy revealed widespread changes in the nervous system. In one, a woman of forty-eight, an acute multiple neuritis was accompanied by delirium, hallucinations, and romancing, with, later, convulsions, muscular twitchings, and spasticity. Arteriosclerosis and fatty liver were present, with degeneration of many peripheral nerves, and of the posterior columns, and direct cerebellar tracts, together with changes in the spinal cornual and the cortical cells. The second case, a man of thirty-five, presented chronic neuritis, mental confusion, irritability, amnesia for recent events, and mild delirium. Post-mortem fatty degeneration and arteriosclerosis were again found, together with similar cerebral and spinal changes to those observed in the first case. The association of mental symptoms with alcoholic neuritis is common, and the chief interest of these two cases lies in the detailed account of the naked eye and microscopic changes found in the brain and spinal tracts. Under the somewhat sensational title of "Soul paralysis," a term first used by Munk, a case has been recorded by H. H. Hoppe,[20] similar to a few other cases reported by Bruns and others, in which a curious loss of voluntary and spontaneous motor power was observed. In these patients the paralysis is not due to actual motor defect, but results from the cutting off of sensory stimuli, so that certain movements can be imitated, but not initiated. An appearance of idiocy or imbecility may also be simulated by the presence of such defects as sensory aphasia, paraphasia, agraphia, or alexia. The symptoms are somewhat variable, but the pathological condition found is fairly uniform, and consists in destruction of the cortex or subcortical region of the parietal or temporo-sphenoidal lobes or of the sensory tracts. Similarly a false appearance of intellectual defect may be apparent in children, the subjects of congenital word blindness and congenital word deafness. Such cases are comparatively rare, but attention has recently been drawn to them by C. J. Thomas,[21] of the Education Department of the London County Council.

[15] Med. Rec., July 22. [16] Clin. Jour., June 7. [17] Med. Rec., July 22, p. 162. [18] Jour. Nerv. and Ment. Dis., April, p. 257. [19] Ibid., March. [20] Ibid. [21] Lancet, June 3, p. 1527.

